# Synthesis of Core–Shell Polyborosiloxanes as a Heat-Resistant Platform

**DOI:** 10.1021/acsomega.2c05056

**Published:** 2022-11-23

**Authors:** Deniz Gunes, Bunyamin Karagoz

**Affiliations:** † Department of Chemistry, Istanbul Technical University, Maslak, 34469 Istanbul, Turkey; ‡ Denge Kimya ve Tekstil San. Tic. A.S, Velimese OSB Mah. 259. Sk. No:4/1, Ergene, 59880 Tekirdag, Turkey

## Abstract

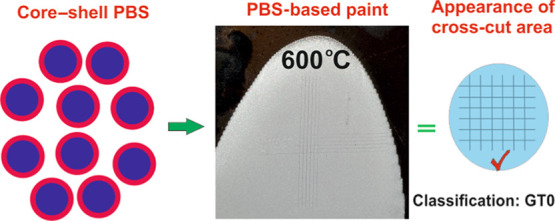

Herein, new polyborosiloxanes (PBSs) were prepared using a straightforward synthetic approach to obtain a core–shell structure as a material with various features such as better adhesion ability to the applied surface and enhanced thermal properties. In this concept, in situ core–shell formation was allowed by sequential addition of ingredients with fixed conversions. First, pre-condensed polysiloxane was synthesized, with a 60% conversion, as a core by the reaction of phenyltriethoxysilane in the presence of water in an acidic condition. Subsequent addition of boric acid into the pre-condensate and a further condensation reaction resulted in the formation of the shell layer through the introduction of the −Si–O–B– bonds to the network of the PBS. The resulting resin was used as a binder for heat-resistant paint in combination with an aluminum pigment, and the paint applied on a metal plate was found to be resistant up to 600 °C in terms of adhesion strength. It was also demonstrated that the incorporation of boron in the core–shell structure showed better adhesion strength than the one-pot preparation of PBS. Using this method, not only the heat resistance requirement of the industrial coating was achieved but also the flame-retardant ability was introduced.

## Introduction

In recent years, boron- and silicon-containing polymers, namely, polyborosiloxanes (PBSs) have been extensively studied due to their heat resistant,^1−6^ flame retardant,^7−9^ ceramic forming,^10,11^ and self-healing^12−14^ properties. The latter is due to the nature of the −B–O–Si– bond in the network structure, which splits and combines reversibly due to moisture susceptibility. On the other hand, the high bonding energies of B–O and Si–O, 537.6 kJ/mol and 460.5 kJ/mol, respectively, boost the thermal properties in combination with the glassy film formation of boron compounds.^1,7^ Another factor affecting the thermal stability is the organic substituent of the Si–R bond, where R is usually selected from the methyl or phenyl groups due to their long half-life time at 250 °C, which are approximately 14 months and 11 years, respectively.^15^ As mentioned briefly above, it was the aim to prepare phenyl-containing PBS resin due to its unique properties. Several “one-pot” methods have been described for PBS preparation in the literature using silanes and various boron sources such as boric acid,^1,2,5,9,10^ trialkyl borate,^16,17^ and borohydride.^14,18^ Linear or partially crosslinked PBSs were also prepared by reacting polydimethylsiloxane derivatives with sodium tetraborate^12^ and/or boric acid.^13,19^ Apart from the above-mentioned routes, our strategy is to prepare PBS as an outer layer of polysiloxane resin, which has been proposed for the first time, to be used as a heat-resistant binder.

Heat-resistant paints mainly composed of methyl/phenyl substituted silicon resin as a binder and thermally stable pigments such as aluminum or iron oxide are used as heat-resistant coatings up to 600–650 °C especially for metal surfaces such as stoves, chimneys, and heat exchangers.^20^

As an important novel method, we have described PBS resins composed of polysiloxane as a core encapsulated by a PBS shell. PBS along with an aluminum pigment was incorporated into a heat-resistant paint formulation and coated on a metal panel with a 35 μm dry film thickness. The results showed that the adhesion strength of the coatings held up to 600 °C for 2 h, which was quite satisfactory as GT0. We characterized the resins using Fourier transform infrared (FTIR) spectroscopy, thermogravimetric analysis (TGA), NMR, scanning electron microscopy (SEM), transmission electron microscopy (TEM), and X-ray photoelectron spectroscopy (XPS) analysis.

## Results and Discussion

### Design and Characterization of the PBS

**Scheme 1:**
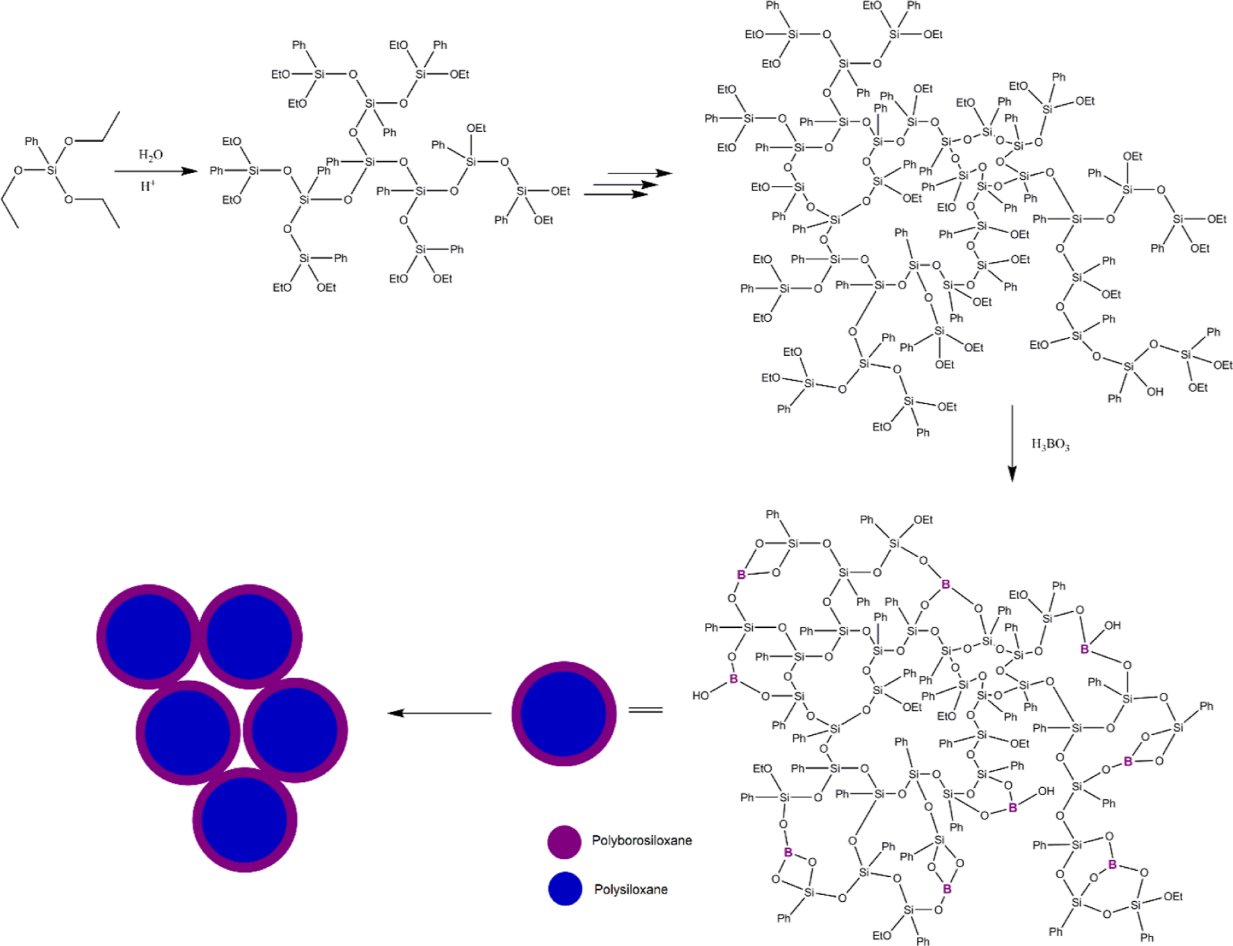
Schematic Illustration of PBS in a Core–Shell Structure

A small manipulation of the synthetic processes during the preparation of polymers allowed many opportunities for designing the polymeric network structure. In our case, addition of the reagent during the condensation reaction in two steps resulted in the formation of a polysiloxane core and a PBS shell layer as a novel strategy, different from the commonly employed one-pot route in the literature for randomly distributed PBS. From the view of the structural aspect, this minor change core network was formed through −Si–O–Si– linkages, whereas the shell was formed through −Si–O–B– and −Si–O–Si– linkages (Scheme 1).

**Figure 1:**
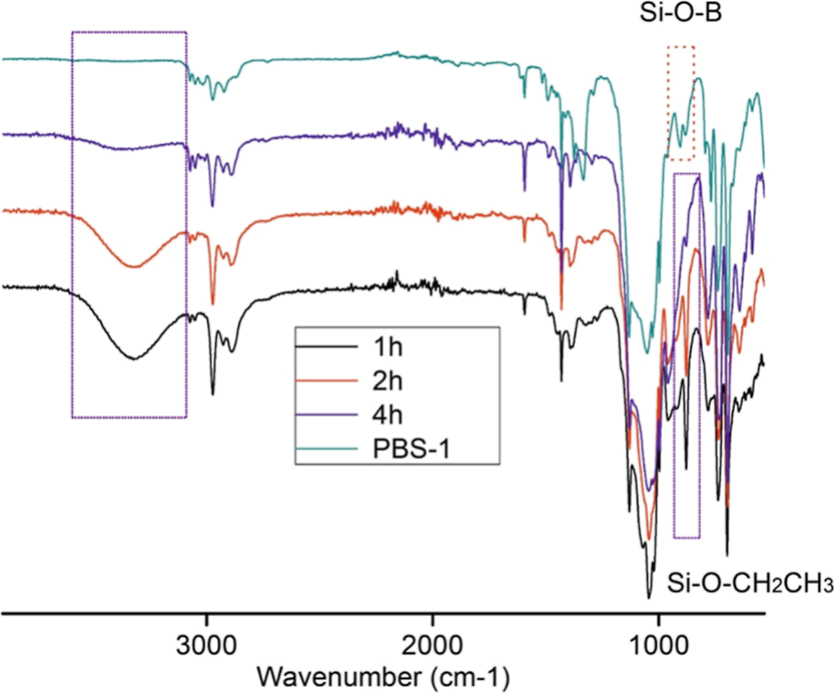
FTIR spectrum of PBS-1 and time-dependent reaction progress.

The FTIR spectrum of the PBS resin is presented in Figure 1 to demonstrate the progress of the reaction and functional moieties in the network. During the synthesis, the Si–O–CH_2_CH_3_ peak at 960 cm^–1^ and the −OH vibration signal at 3400 cm^–1^ disappear with time, which indicates that the condensation between alkoxy silane and hydroxy sources takes places. Classical Si–O–Si and B–O signals are observed at around 980–1130 and 1350–1450 cm^–1^, respectively. The peaks appearing at 670 and 905 cm^–1^ were associated with Si–O–B bonds, which confirms the formation of PBS.^9^ C=C vibration peaks at 1600 cm^–1^ and C–H vibration peaks at 3020–3070 cm^–1^ are due to the phenyl groups of the solvent and silane reagent. Moreover, aromatic C–H bending vibrations belonging to phenyl groups appeared at 881 cm^–1^.

**Figure 2:**
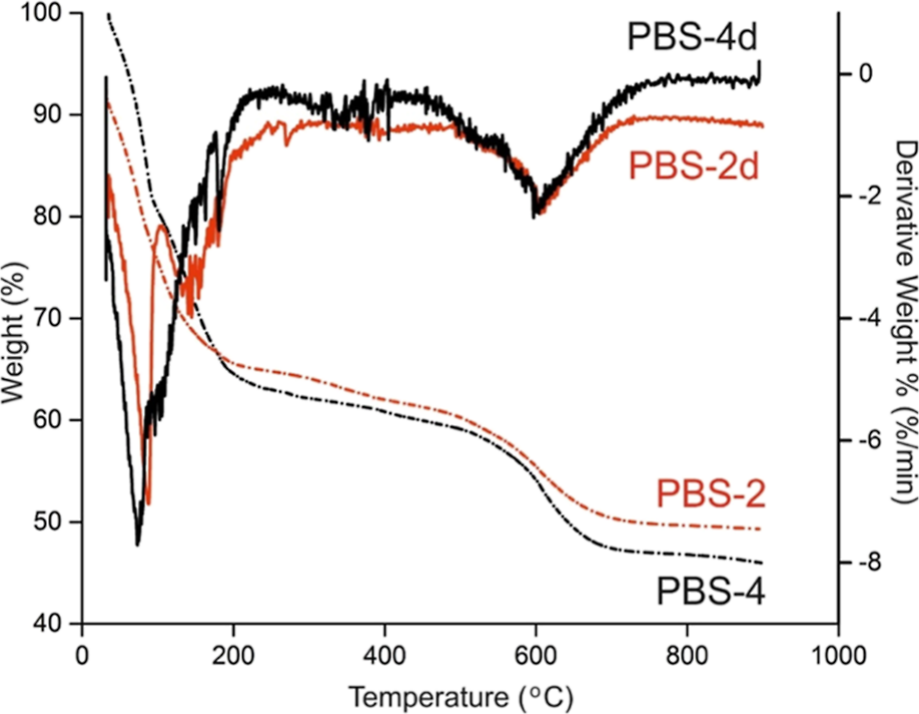
TGA thermograms of PBS-2 and PBS-4.

TGA performed under a nitrogen atmosphere shows that the solvent is evaporated at around 120–150 °C (Figure 2). A new sharp peak in the derivative of the PBS-2 thermogram between 150 and 250 °C is attributed to the Si–O–B bond cleavage due to the in situ generation of silanol groups under heating, which makes sense considering the boron ester shell structure of the material. PBS, on the other hand, is stable up to 500–600 °C and is in the maximum deterioration phase at 620 °C. PBS gives an almost 50% charry residue at 900 °C, which is compatible with the theoretical calculation. Furthermore, improvement of thermal properties of the boron-incorporated polyphenylsiloxane matrix has been proved by TGA, where the residual content was doubled at 900 °C, as given in Figure S1.

^11^B NMR was performed to observe the boron linkage characteristic in PBS-2. There appeared one broad peak at 18.7 ppm, which indicated the formation of both B(OSi)_3_ and B(OSi)_2_OH structures (Figure S2). SEM–energy-dispersive X-ray spectroscopy (EDX) and SEM images of the PBS-1 and PBS-2 resins were obtained for observing the core–shell structure of the boron-based resins. No meaningful results were obtained (Figures S3 and S4).

**Figure 3:**
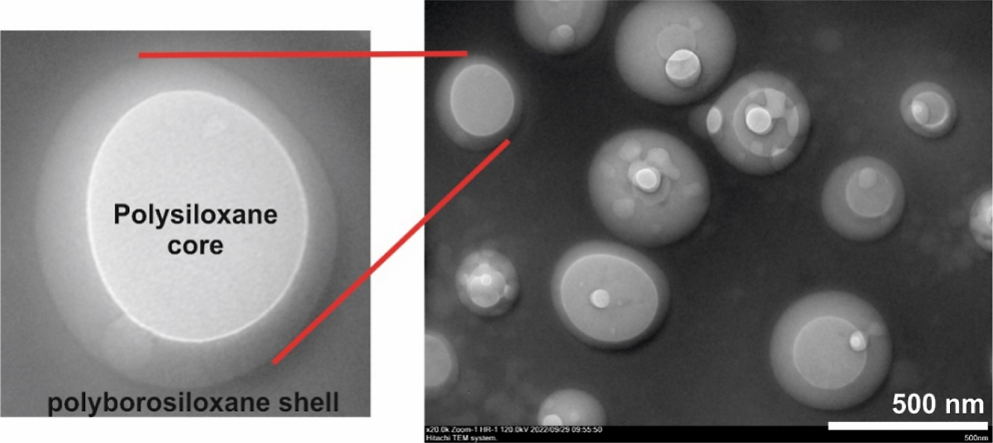
TEM images of the PBS-2 sample.

The morphology and core–shell structure of the resulting PBSs (PBS-1 and PBS-2) were investigated via TEM images, as shown in Figures 3 and S5. First, the xylene solution containing 65 wt % PBS was dropped onto the copper grid, and then, TEM images were obtained from the dried sample. The images revealed the formation of the core–shell structure via color contrast between the polysiloxane core and PBS shell. The core–shell structures were irregular, as expected.

**Figure 4:**
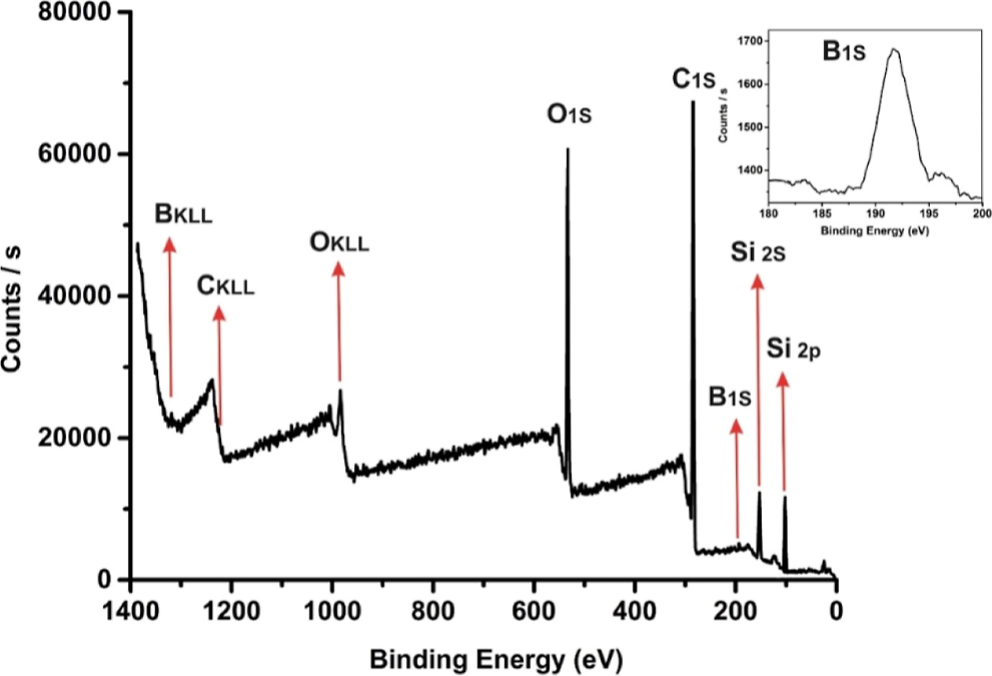
XPS survey scan spectrum of the PBS-2 sample.

XPS was used to determine the chemical states of the PBS samples PBS-1 and PBS-2 (Figures 4 and S6). In the spectrum, it was observed that the PBS-2 sample surface consists of O, C, B, and Si with binding energies of O 1s, C 1s, B 1s, Si 2s, and Si 2p at 533.1, 284.5, 191.63, 153, and 102 eV, respectively. The B 1s peak region revealed the B–O–Si and B–OH bond existence at the surface of the PBS sample.^10,21^ The C 1s peak area of the PBS-2 sample proved the presence of three chemical states, corresponding to C–Si, C–C/C=C, and C–O bonds. Two binding energies were observed at 532.22 and 533.65 eV, attributed to Si–OH and Si–O–Si bonds, respectively, for the O 1s peak (Figure S7).

### Heat-Resistant Performance of PBS

The main concept of the heat-resistant paint is to be stable up to a required temperature such as 400 °C for decorative paints and 600 °C for industrial paints. Additional properties such as corrosion resistance or flame retardancy may also be demanded depending on the final application. Our motivation was to invent not only heat-resistant but also flame-retardant resin in one product. The latter is a natural consequence of possessing boron in the structure. To understand in a better way, PBS-2 was dried at room temperature and subjected to a continuous flame, and quenching of the flame was noted right after the removal of the flame source, while the commercial product keeps burning.

**Figure 5:**
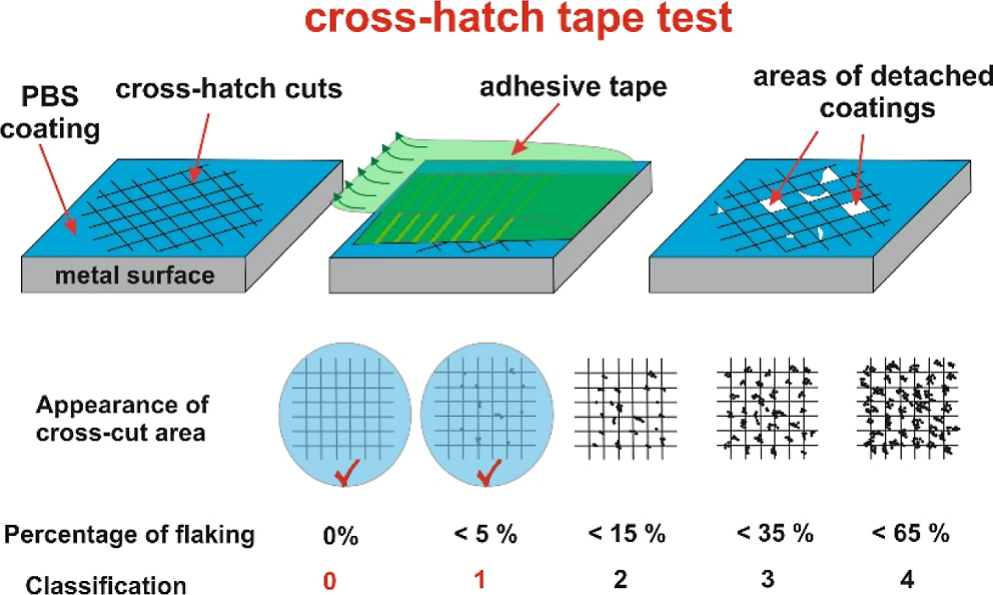
Cross-cut test method and classification of adhesion strength.

In terms of heat-resistant properties or adhesion strength, it was found that the resin type, boron content, film thickness, and application temperature play important roles. Adhesion strength is measured by a cross-hatch tape test, as given below in Figure 5, which shows that the better the adhesion strength is the less detached the areas are after the removal of the adhesive tape. In this concept, GT0 classification is the best result.

**Figure 6:**
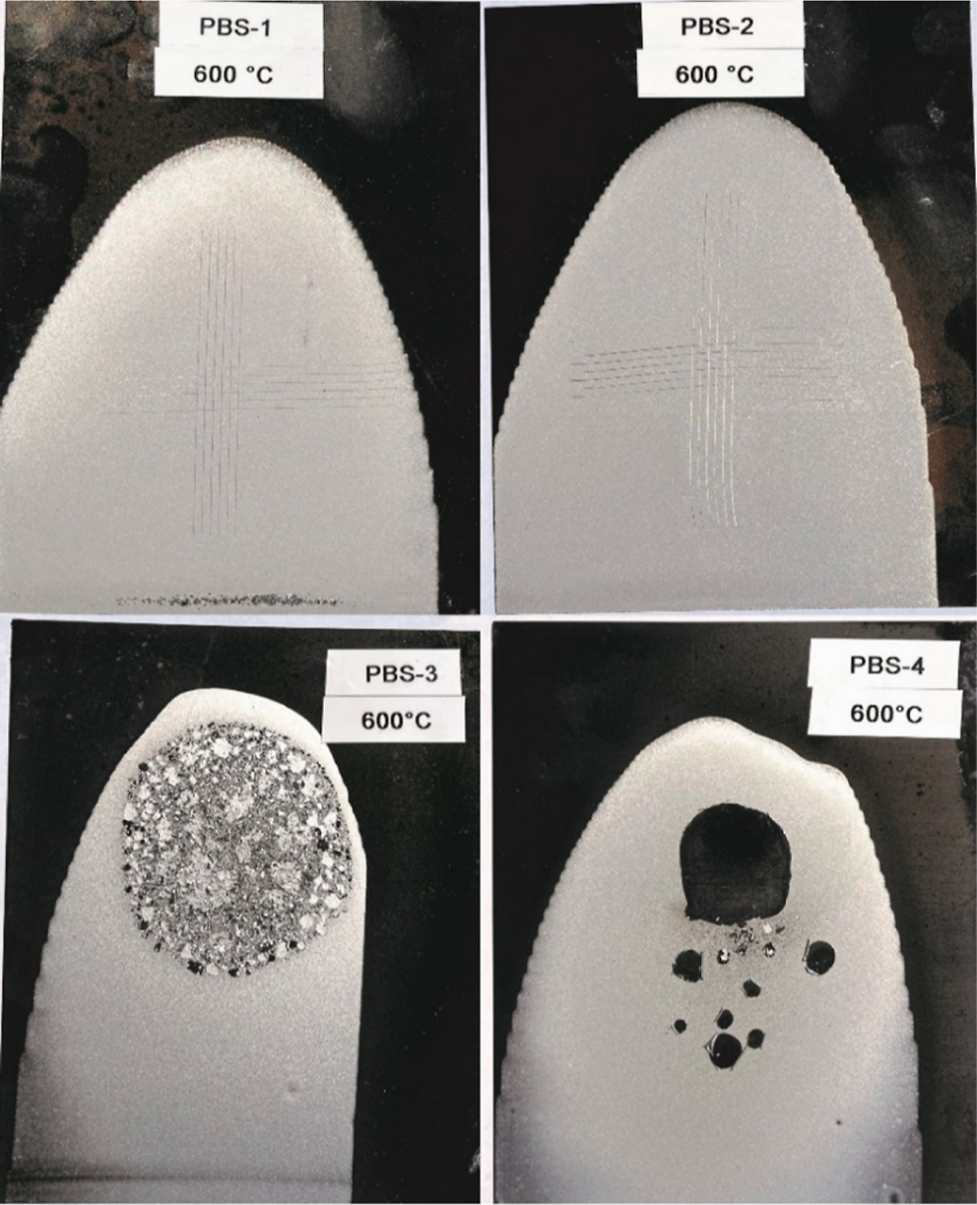
PBS coatings held at 600 °C for 2 h and image of cross-cut if applicable.

It is important to note that core–shell structures were demonstrated to be resistant up to 600 °C as GT0–GT1 even though the boron content was low (see Table 1 and Figure 6), while one-pot PBS resulted in a separation from the surface at the same temperature. As previously mentioned, it is observed through TGA that heating the PBS splits the Si–O–B bonds and creates Si–OH bonds in the PBS network, which readily react with the pigment and metal surface at the same time. From a morphological point of view, the core–shell provides bulk binding sites that increase the adhesion strength.

**Table 1:** Adhesion Strength of PBS-Containing Heat-Resistant Paints at High Temperatures

PBS resin	resin type	% boron content	400 °C	600 °C
PBS-1	core–shell	0.64	GT0	GT0
PBS-2	core–shell	1.22	GT0	GT0
PBS-3	one-pot	0.64	n.a^a^	n.a^a^
PBS-4	one-pot	1.22	GT0	n.a^a^

a Cross-cut is not applicable due to the big separations.

**Figure 7:**
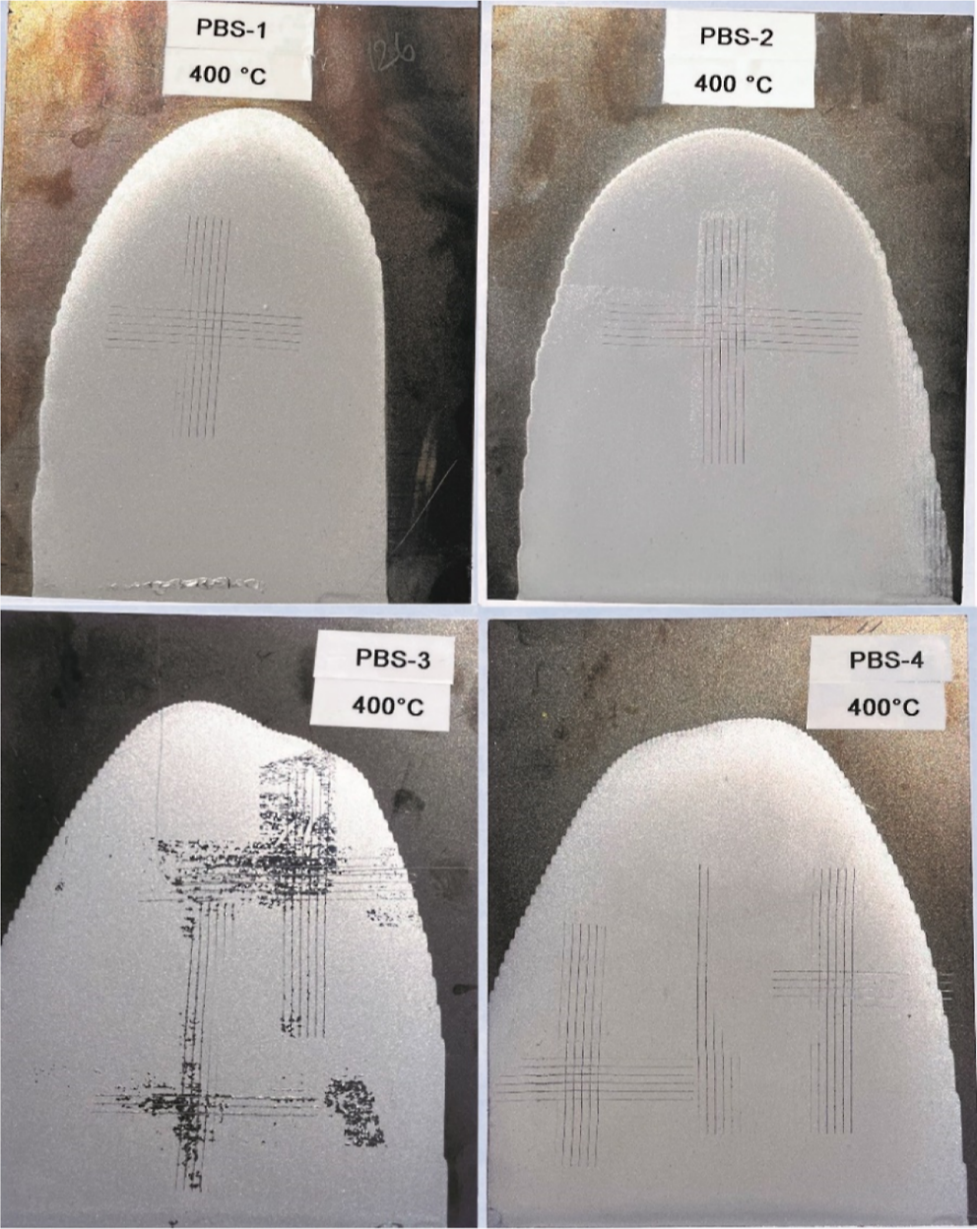
PBS coatings held at 400 °C for 2 h and image of cross-cut if applicable.

On the other hand, PBS-4 was found to be resistant up to 400 °C, which was attributed to the increased binding sites compared to that of PBS-3 (Figure 7). In terms of film thickness, all the good results were obtained at a 100 μm wet film thickness and, an increment of the film thickness to 200 μm resulted in no adhesion to the metal surface.

## Conclusions

In summary, we have prepared PBS resins composed of polysiloxane as a core encapsulated by a PBS shell for the first time. PBS as a combined product has not only enhanced the thermal stability but also flame-retardant properties. PBS prepared via a core–shell strategy was found to be an excellent platform for heat-resistant coating applications up to 600 °C. It was also revealed that increasing the binding sites increased the adhesion. We believe that our study can open new paths toward designing innovative coatings. In a future work, the flame retardant property of PBS will be investigated.

## Experimental Section

### Materials

Phenyltriethoxysilane (PTES), boric acid (Eti Maden), sulfuric acid, xylene, non-leafing aluminum pigment paste with 65% active content (Eckart), and BENTONE 038 (Elementis) paste with 12% active content were supplied by Denge Kimya, and all the chemicals were of industrial grade and not less than 97% purity.

### Preparation of PBSs

#### PBS-1

61.7 g of PTES (256.7 mmol) and 0.1 g of sulfuric acid (1.02 mmol) were mixed at room temperature for 10 min in a 100 mL round-bottom flask, and then, 6.4 g of water (355.5 mmol) was added dropwise. The mixture was stirred overnight, and ethanol was distilled off as a side product at 95 °C. During distillation, 2 g of boric acid (32.3 mmol) and 8.5 g of xylene were added to the reaction medium at 57 and 74% yields, respectively. Distillation was completed up to 90% yield at 115 °C under 0.5 bar vacuum. The final product was diluted with xylene/isobutanol to give a clear solution and 65% solid content, and the catalyst was neutralized with the required amount of sodium bicarbonate at room temperature.

#### PBS-2

61.7 g of PTES (256.7 mmol) and 0.1 g of sulfuric acid (1.02 mmol) were mixed at room temperature for 10 min in a 100 mL round-bottom flask, and then, 5.37 g of water (298.3 mmol) was added dropwise. The mixture was stirred overnight, and ethanol was distilled off as a side product at 95 °C. During distillation, 3.9 g of boric acid (63.07 mmol) and 8.5 g of xylene were added to the reaction medium at 46 and 82% yields, respectively. Distillation was completed up to 92% yield at 115 °C under 0.5 bar vacuum. The final product was diluted with xylene/isobutanol to give a clear solution and 65% solid content, and the catalyst was neutralized with the required amount of sodium bicarbonate at room temperature.

#### PBS-3

61.7 g of PTES and 0.1 g of sulfuric acid were mixed at room temperature for 10 min in a 100 mL round-bottom flask, and then, 6.4 g of water (dropwise) and 2 g of boric acid were added into the flask. The mixture was stirred overnight, and ethanol was distilled off as a side product at 95 °C. In order to prevent gelation, 10 and 5 g of xylene were added to the reaction medium at 64 and 76% yields, respectively. Distillation was completed up to 92% yield at 115 °C under 0.3 bar vacuum. The final product was diluted with xylene/isobutanol to give a clear solution and 65% solid content, and the catalyst was neutralized with the required amount of sodium bicarbonate at room temperature.

#### PBS-4

61.7 g of PTES and 0.1 g of sulfuric acid were mixed at room temperature for 10 min in a 100 mL round-bottom flask, and then, 5.37 g of water (dropwise) and 3.9 g of boric acid were added into the flask. The mixture was stirred overnight, and ethanol was distilled off as a side product at 95 °C. In order to prevent gelation, 10 and 5 g of xylene were added to the reaction medium at 65 and 74% yields, respectively. Distillation was completed up to 90% yield at 115 °C under 0.4 bar vacuum. The final product was diluted with xylene/isobutanol to give a clear solution and 65% solid content, and the catalyst was neutralized with the required amount of sodium bicarbonate at room temperature.

#### Phenylpolysiloxane

61.7 g of PTES and 0.1 g of sulfuric acid were mixed at room temperature for 10 min in a 100 mL round-bottom flask, and then, 5.37 g of water was added dropwise. The mixture was stirred overnight, and the catalyst was neutralized. 10 g of xylene was added to the reaction medium when 22 g of ethanol was distilled off at 95 °C. Distillation was completed up to 90% yield at 115 °C under vacuum. The final product was diluted with xylene to give a 65–70% solid content.

### Preparation and Application of Heat-Resistant Paint with PBS

Heat-resistant paint containing PBS was prepared using a guide formulation given in Table 2 where PBS is a binder, BENTONE 38 is a thickener, aluminum is used as a heat-resistant pigment, and xylene is used as the solvent. All ingredients were mixed and stirred for 30 min using a mechanical stirrer, and then, the mixture was applied using an automatic film applicator (according to ASTM D823) to give 100 and 200 μm wet film thicknesses on a 10 × 20 cm^2^ metal plate ,which was cleaned with xylene and dried prior to the application.

**Table 2:** Heat-Resistant Paint Formulation

ingredient	%
PBS	40
BENTONE 38 paste	8
aluminum paste (non-leafing)	12
xylene	40

The painted metal plates reached tack-free time in less than an hour at room temperature and then were cured at 200 °C in a muffle furnace for 30 min. Afterward, the samples were kept at 400 °C for 2 h and cooled down to room temperature, and a cross-cut test was performed to measure the adhesion strength. Likewise, different plates were prepared at 600 °C, and the same procedure was repeated.

### Characterization

FTIR spectra were obtained using a Thermo Scientific Nicolet iS50 FTIR Spectrometer. TGA was carried out under a nitrogen atmosphere using a PerkinElmer Diamond TGA 4000 instrument at a heating rate of 20 °C/min till 900 °C. ^11^B NMR spectra of the samples were recorded by using CDCl_3_ as a solvent on a Bruker Biospin AVANCE III-400 MHz spectrometer. An FEI Quanta FEG 250 FE-SEM instrument was used to obtain information about the morphology of the PBS. The surface structures of the samples were determined using a high-resolution transmission electron microscope (Hitachi 7700) equipped with an acceleration voltage of 120 kV. XPS was performed using an XPS system (Specs, Flex-XPS) with a monochromatic Al K X-ray source. The range of the binding energy was 0–400 eV.

## Supporting Information Available

TGA, ^11^B NMR, SEM, SEM–EDX, TEM, and XPS characterizations of PBS (PDF)
