# Supplementary material for: Synthesis of Core–Shell Polyborosiloxanes as a Heat-Resistant Platform
Source: ACS Omega. 2022 Nov 23;7(48):43877–82. doi: 10.1021/acsomega.2c05056 (PMC9730314; doi:10.1021/acsomega.2c05056)
Supplement: Supplementary file 1 — ao2c05056_si_001.pdf [file ao2c05056_si_001.pdf]

Supporting Information

Synthesis of Core-Shell Polyborosiloxanes as Heat Resistant Platform

Deniz Gunes[a,b], Bunyamin Karagoz[b*]

Deniz Gunes – Department of Chemistry, Istanbul Technical University, Maslak, 34469, Istanbul, Turkey, email:denizsun@gmail.com; Denge Kimya ve Tekstil San. Tic. A.S, Velimese OSB Mah. 259. Sk. No:4/1 Ergene, 59880 Tekirdag, Turkey; email:dgunes@dengekimya.com

Bunyamin Karagoz – Department of Chemistry, Istanbul Technical University, Maslak, 34469, Istanbul, Turkey; email:karagozb@itu.edu.tr

*Correspondig author

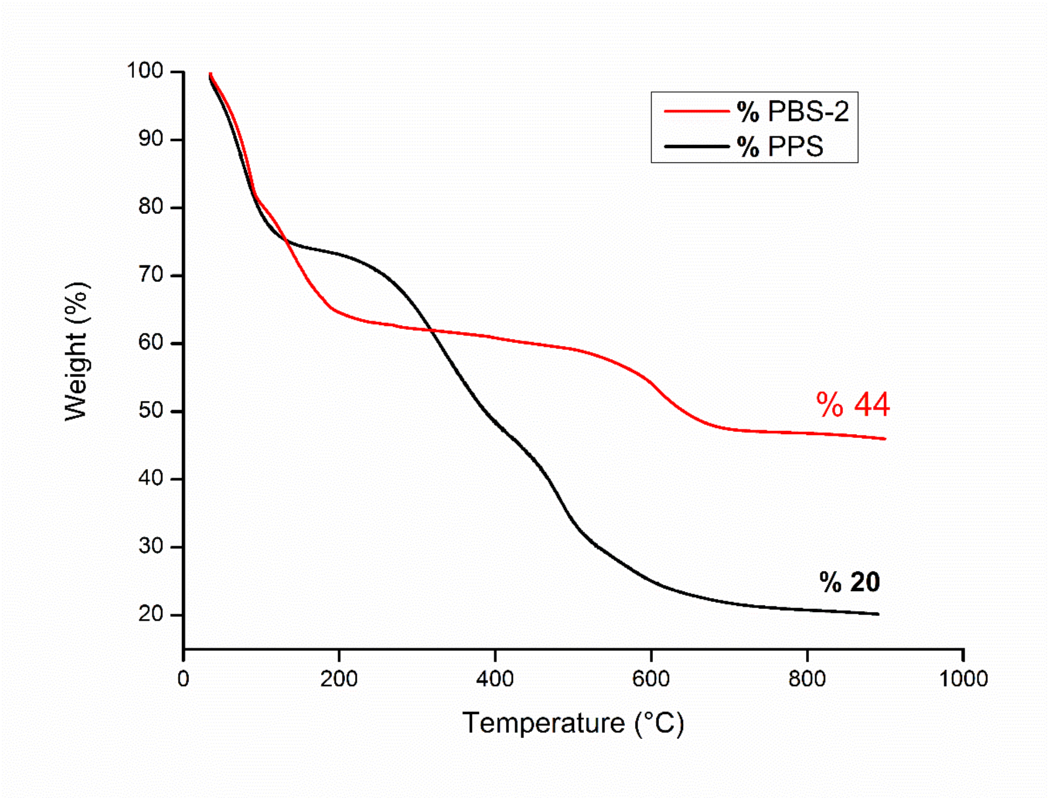

Figure S1. TGA thermogram of the polyborophenylsiloxane and polyphenylsiloxane

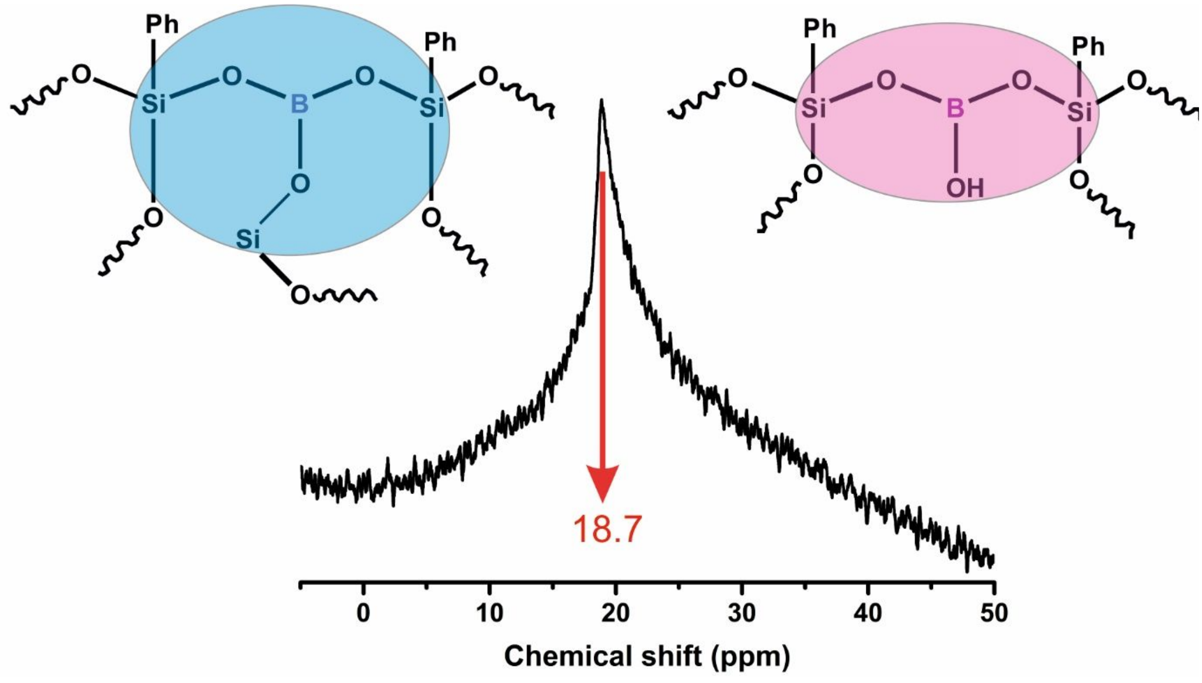

Figure S2.^{11}B NMR spectrum of PBS-2

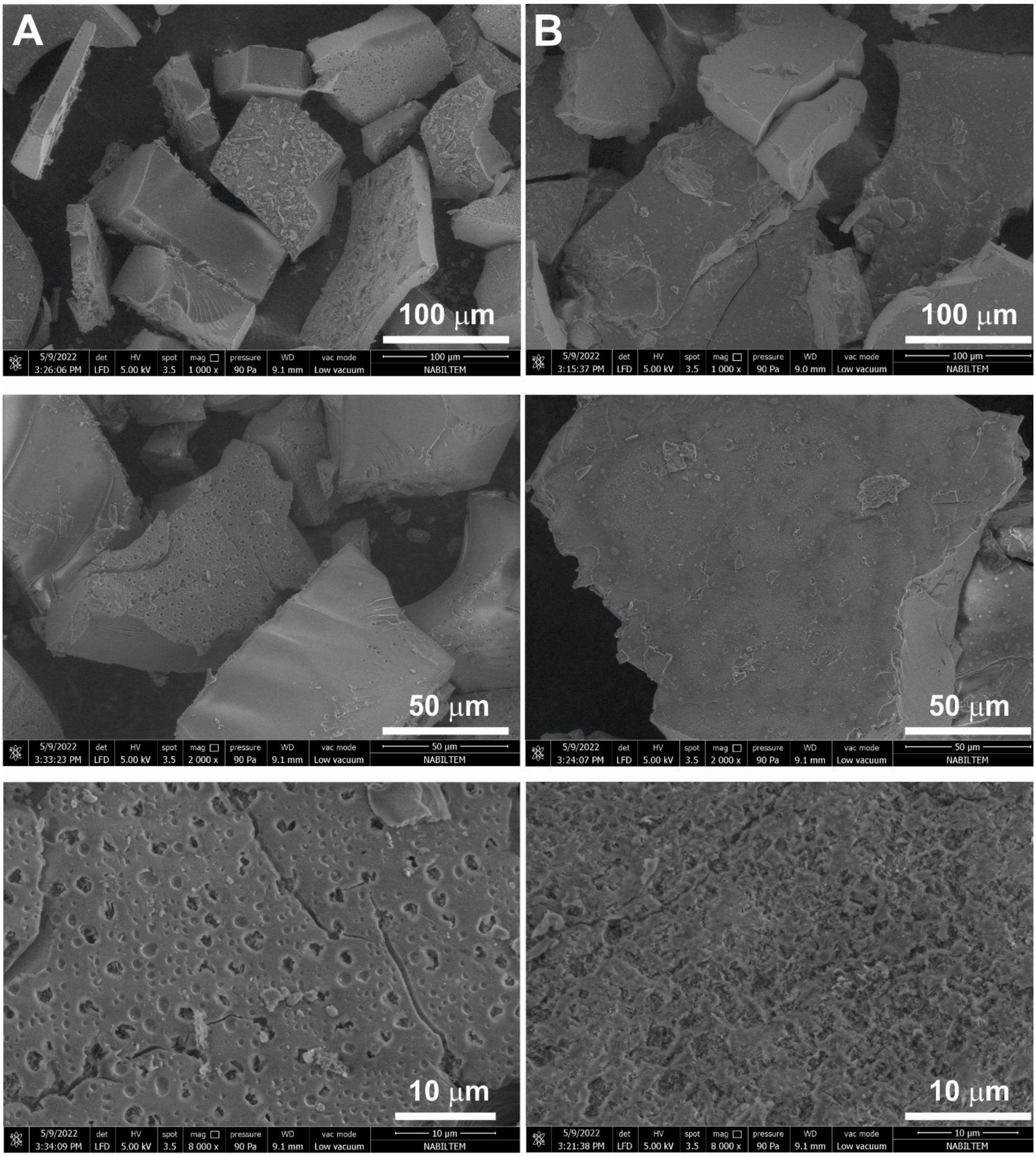

Figure S3. SEM images of PBS-2 (A) and PBS-1 (B) dried at ambient temperature.

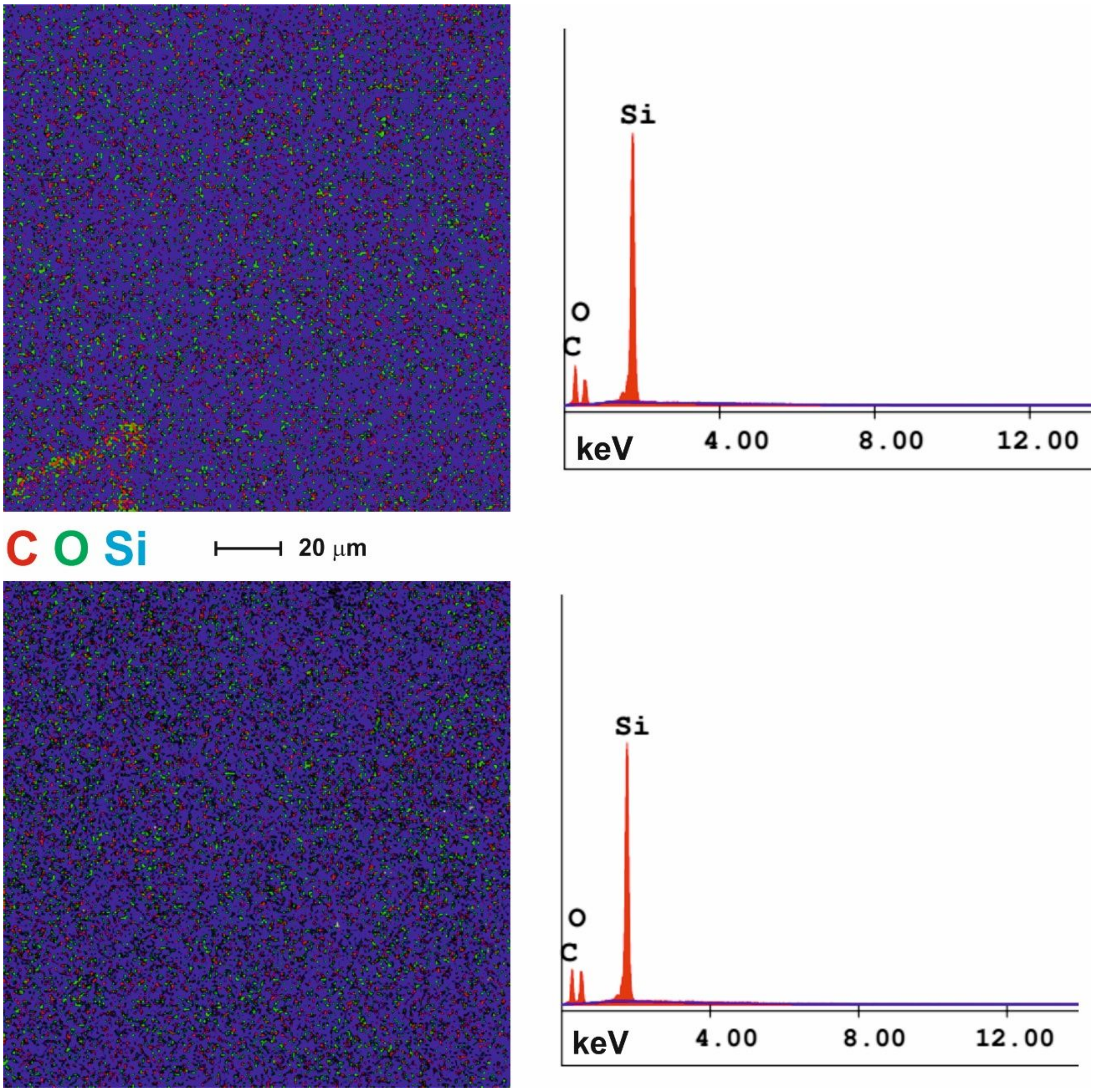

Figure S4. SEM-EDX analysis of PBS-2 resin

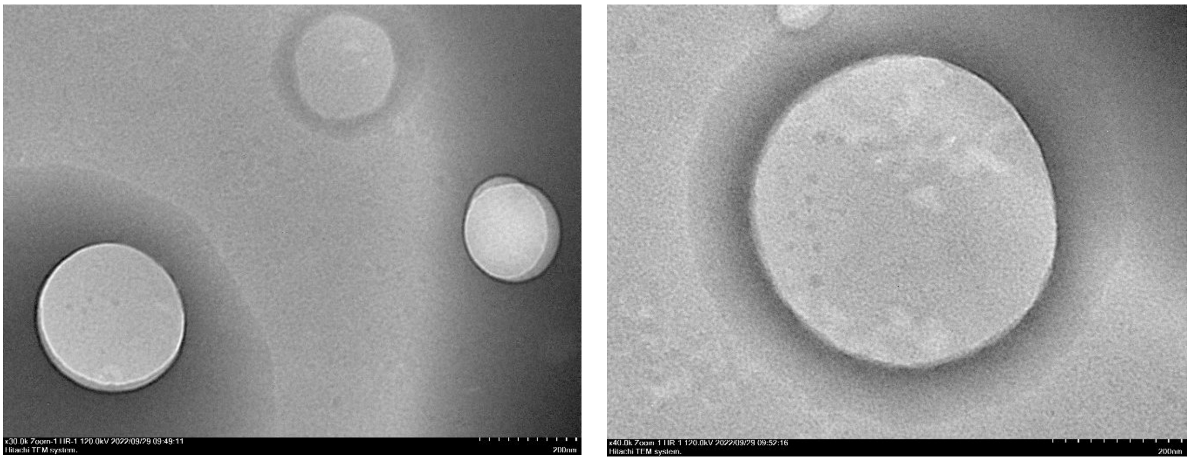

Figure S5. TEM images of PBS-1

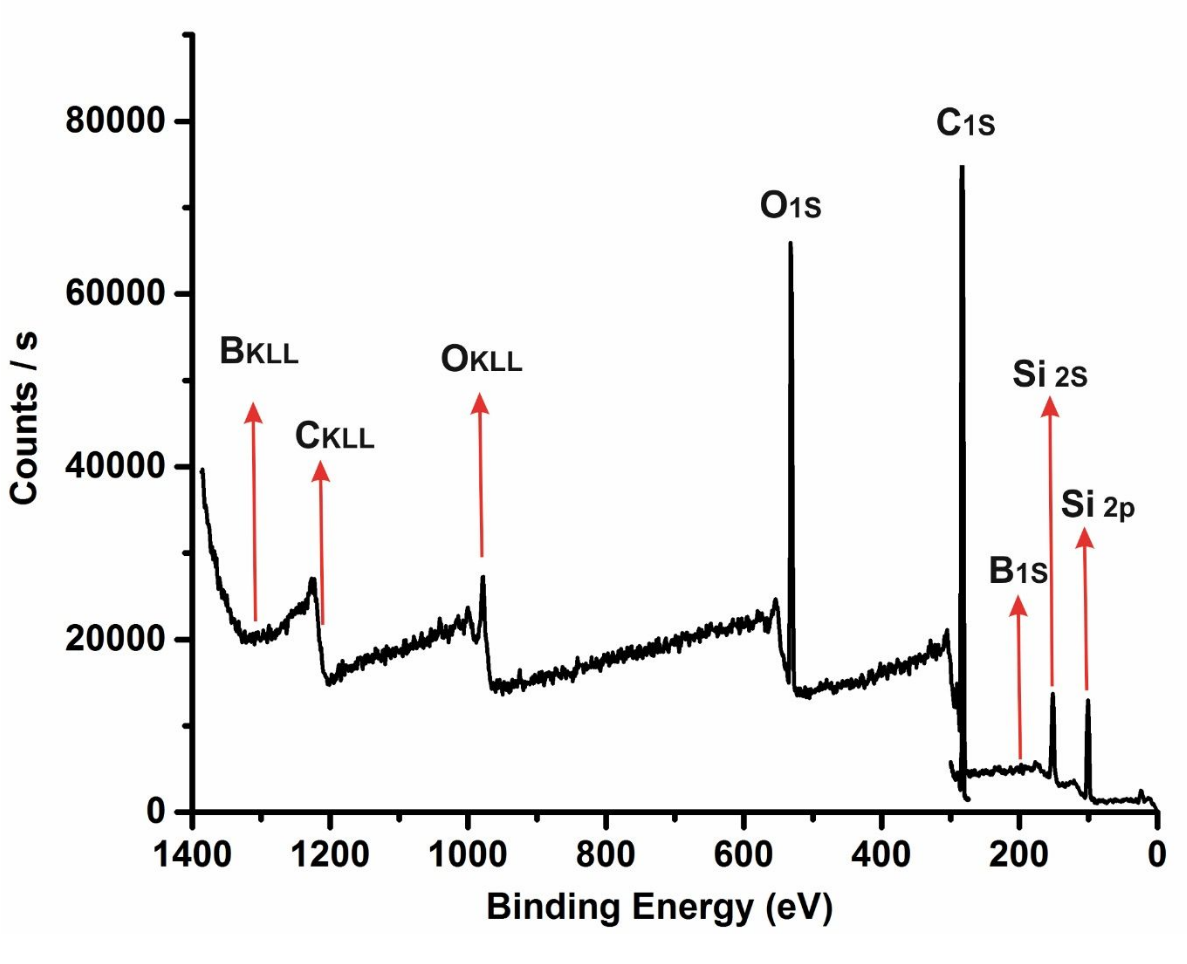

Figure S6: XPS survey of the PBS-1 sample

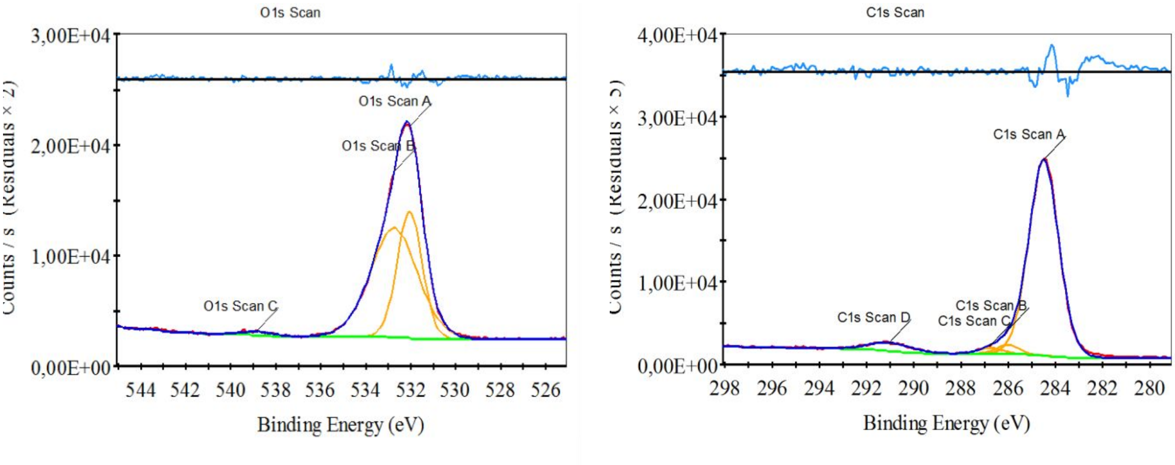

Figure S7: XPS spectra of the O 1s and C1s of the PBS-2 sample
